# Epidurals for Coarctation Repair in Children Are Associated with Decreased Postoperative Anti-Hypertensive Infusion Requirement as Measured by a Novel Parameter, the Anti-Hypertensive Dosing Index (ADI)

**DOI:** 10.3390/children6100112

**Published:** 2019-10-10

**Authors:** J. Matthew Kynes, Matthew S. Shotwell, Camila B. Walters, David P. Bichell, Jason T. Christensen, Stephen R. Hays

**Affiliations:** 1 Department of Anesthesiology, Vanderbilt University Medical Center, Nashville, TN 37232, USA; j.matt.kynes@vumc.org (J.M.K.); Stephen-hays@uiowa.edu (S.R.H.); 2 Department of Biostatistics, Vanderbilt University Medical Center, Nashville, TN 37232, USA; matt.shotwell@vumc.org; 3 Department of Surgery, Vanderbilt University Medical Center, Nashville, TN 37232, USA; david.bichell@vumc.org; 4 Department of Pediatrics, Vanderbilt University Medical Center, Nashville, TN 37232, USA; jaschristensen@childrensomaha.org

**Keywords:** analgesia, epidural, anti-hypertensive agents, aortic coarctation, child, hypertension, postoperative period

## Abstract

Background: Sympathetically-associated hypertension after coarctation repair is a common problem often requiring anti-hypertensive infusions in an intensive care unit. Epidurals suppress sympathetic output and can reduce blood pressure but have not been studied following coarctation repair in children. We sought to determine whether epidurals for coarctation repair in children were associated with decreased requirement for postoperative anti-hypertensive infusions, if they were associated with changes in hospital course, or with complications. Methods: In this observational retrospective cohort study, we evaluated all patients age 1–18 years undergoing coarctation repair at our institution during a 10-year period and compared the requirement for postoperative anti-hypertensive infusions in patients with and without epidurals using an anti-hypertensive dosing index (ADI) incorporating total dose-hours of all anti-hypertensive infusions (primary outcome). We also assessed intensive care unit (ICU) and hospital length of stay, discharge on oral anti-hypertensive medication, and complications potentially related to epidurals (secondary outcomes). Results: Children undergoing coarctation repair with epidurals had decreased requirements for postoperative anti-hypertensive infusions compared to children without epidurals (cumulative ADI 65.0 [28.5–130.3] v. 157.0 [68.6–214.7], *p* = 0.021; mean ADI 49.0 [33.3–131.2] v. 163.0 [66.6–209.8], *p* = 0.01). After multivariable cumulative logit mixed-effects regression analysis, mean ADI was decreased in patients with epidurals throughout the postoperative period (*p* < 0.001). Patients with epidurals were 1.6 years older and weighed 10.6 kg more than patients without epidurals but were otherwise comparable. Epidural complications included pruritus (three patients), agitation (one patient), somnolence (one patient), and transient orthostatic hypotension (one patient). Duration of intensive care unit admission, duration of hospital stays, and requirement for anti-hypertensive medication at discharge were similar in patients with and without epidurals. Conclusions: This is the first study of children receiving an epidural for surgical repair of aortic coarctation via open thoracotomy. In this small, single-institution, observational retrospective cohort study, epidurals for coarctation repair in children were associated with decreased postoperative anti-hypertensive infusion requirements. Epidurals were not associated with length of ICU or hospital stay, or with discharge on anti-hypertensive medication. No significant epidural complications were noted. Prospective study of larger populations will be necessary to confirm these associations, address causality, verify safety, and assess other effects.

## 1. Introduction

### 1.1. Coarctation Repair and Hypertension

Repair of aortic coarctation is associated with postoperative hypertension [1] in up to two-thirds of patients [2], and chronic hypertension in up to one-third [3]. Post-coarctectomy hypertension is thought to be mediated primarily by stimulation of aortic afferent sympathetic nerve fibers, inducing short-term increases in plasma catecholamines as well as intermediate-term activation of the renin-angiotensin system [2,4,5,6,7,8]. Although catheter-based approaches to coarctation repair have been developed and decrease such sympathetic activation, four risks of aneurysm formation and restenosis dictate that open surgical repair remains the treatment of choice in most infants and children [9].

Management of hypertension after coarctation repair generally requires intensive care unit (ICU) admission and titration of anti-hypertensive infusions [10,11]. Many interventions for blood pressure management in this setting have been assessed including angiotensin-converting enzyme (ACE) inhibitors [12,13,14], beta (β) blockers [15,16,17], calcium channel blockers [18], dexmedetomidine [19], and nitroprusside [16]. While nitroprusside, esmolol and labetalol are most commonly used, there is no consensus on how best to manage these patients postoperatively [11]. Persistent hypertension may increase ICU length of stay and overall costs of hospitalization.

### 1.2. Coarctation Repair and Epidurals

Regional anesthesia may provide superior analgesia post thoracotomy in the peri operative period [20], improve ventilation [21], decrease chronic pain [22], and is considered safe and effective in the management of pediatric cardiac surgery [23,24]. Coarctation repair is generally performed via open thoracotomy, and is thus particularly amenable to thoracic epidural analgesia [25], which is known to inhibit sympathetic activation [26]. Epidurals have been shown to reduce postoperative serum catecholamine, glucose, and adrenocorticotropic hormone (ACTH) levels more effectively than intravenous opioid in children [27] and modulate the hormonal stress response to surgery in patients of all ages, including after cardiac surgery [28,29,30,31,32]. Thoracic epidural analgesia has even been described as superior to interpleural analgesia following coarctation repair in adults [33,34,35]. Despite the potential benefits of epidurals for coarctation repair in children, we could identify only one case report describing three children with epidurals for coarctation repair [36], and one case report describing a child with an epidural for coarctation repair who developed transient Horner syndrome [37]. In addition, one case report described serratus plane block for repair of coarctation in three pediatric patients [38]. A Cochrane review protocol has been developed to compare epidurals with other analgesic regimens [39]. To our knowledge, no study has systematically assessed epidurals for coarctation repair in pediatric patients. 

### 1.3. Aims and Hypothesis

Given the mechanism of hypertension following coarctation repair and the anti-hypertensive effects of epidurals, we assessed anti-hypertensive infusion requirements after coarctation repair via open thoracotomy in children with or without postoperative thoracic epidural analgesia. We hypothesized that patients with epidurals would have decreased requirements for postoperative anti-hypertensive infusions compared to patients without epidurals. We also assessed ICU and hospital length of stay, discharge on oral anti-hypertensive medication, and complications potentially related to epidurals.

## 2. Materials and Methods

This study was approved by the Vanderbilt University Medical Center Institutional Review Board with waiver of informed consent. This study followed the appropriate EQUATOR (Enhancing the Quality of and Transparency of Health Research) guidelines, including the STROBE (Strengthening the Reporting of Observational studies in Epidemiology) checklist for cohort studies.

This was an observational retrospective cohort study of all children age 1–18 years having surgical coarctation repair at our institution from 2007–2016, identified by billing codes and confirmed with pediatric cardiac surgical staff; medical records before this date were inadequate for meaningful analysis. Infants <1 year of age were excluded, as most at our institution are neonates who generally remain intubated for several days after surgery and rarely receive epidurals. Children with significant comorbid renal and congenital heart disease were also excluded given the additional risk of hypertension and complexity of managing these groups. No other major health problems such as pulmonary disease or neurologic disease were present in the patients included in the study. Baseline characteristics included age and weight at surgery, sex, treatment with oral anti-hypertensive medication prior to surgery, and measures of coarctation severity including systolic hypertension z-score [40], upper-extremity to lower-extremity systolic blood pressure (SBP) gradient, and aortic isthmus z-score [41].

The primary outcome was a postoperative anti-hypertensive infusion requirement. No validated score is available to assess this, and therefore a metric incorporating dose and duration of all anti-hypertensive infusions was devised, the anti-hypertensive dosing index (ADI). ADI was quantified by determining the dose of each anti-hypertensive infusion, expressed as a multiple of the standard initial dose, multiplied by the total duration of each infusion at each dose. ADI thus calculated total dose-hours of all anti-hypertensive infusions, analogous to minimum alveolar concentration (MAC)-hours of anesthetic exposure [42]. The standard initial dose of each anti-hypertensive agent was defined from published recommendations [43] as follows: nicardipine = 1 mcg·kg^−1^·min^−1^; esmolol = 100 mcg·kg^−1^·min^−1^; nitroprusside = 0.5 mcg·kg^−1^·min^−1^. These starting doses were used to standardize across medications and that dose amount was quantified in multiples of the starting dose for the corresponding drug. For example, a patient receiving esmolol (standard initial dose = 100 mcg·kg^−1^·min^−1^) at 200 mcg·kg^−1^·min^−1^ for 2 h and 400 mcg·kg^−1^·min^−1^ for 4 h would have an ADI of (2 × 2 h) + (4 × 4 h) = 4 + 16 = 20. Only these three medications were used as infusions for blood pressure control. Cumulative ADI represented the sum of the ADIs of each infusion used. Dosing levels were assessed at ICU admission and every hour thereafter until discontinued. Sedative infusions, including dexmedetomidine and propofol, were not included in the ADI, but were included categorically in regression analysis. 

Secondary outcomes included ICU length of stay (hours), hospital length of stay (days), and hospital discharge on oral anti-hypertensive medication. Complications potentially related to epidurals were noted. Consistent pain scores were not readily available for comparison between groups. Intraoperative hemodynamics were not compared, as epidural infusions were initiated postoperatively.

Patient population, baseline characteristics, primary outcome including ADI, and secondary outcomes were all chosen and defined before data extraction and subsequent statistical analysis. Given the observational retrospective cohort study design using a novel metric (ADI) as primary outcome, sample size and power analysis were not calculated a priori.

### Statistical Analysis

Patient characteristics were compared between groups using the Pearson chi-square test or Wilcoxon rank sum test as appropriate. 

The covariate-adjusted effect of intervention on the time course of mean ADI following surgery was quantified using cumulative logit mixed-effects regression, adjusting for pre-operative anti-hypertensive use, SBP gradient, hypertension z-score, aortic z-score, patient body mass, and sex. A random intercept term was used to account for heterogeneity among patients or, equivalently, to account for the within-subject correlation among serial observations [44]. This method treats the outcome, ADI, as an ordinal random variable, but is suitable for regression of quantitative outcomes. In order to facilitate this approach, we rounded each ADI measurement to the nearest integer. Time following surgery was modeled using a five-knot natural spline function to account for nonlinear effects, where the knots were assigned evenly across the quantiles of the time variable. The intervention effect was modeled as an interaction between the time effect and epidural use. Adjustment variables were modeled in a linear fashion, and no additional interactions were considered. The adjusted effect of intervention was summarized graphically by plotting the mean ADI over time, with 95% confidence interval, for patients with epidurals versus patients without epidurals, adjusted for the typical patient (i.e., at the median or mode of each adjustment variable). The overall statistical significance of the intervention effect was assessed using a multiple-degree-of-freedom test on the coefficients associated with the time-by-intervention interaction. *P*-values < 0.05 were considered statistically significant.

## 3. Results

### 3.1. Baseline Characteristics

A total of 46 patients were identified: 15 had thoracic epidurals for postoperative analgesia placed in the operating room under general endotracheal anesthesia after completion of the surgical procedure; 31 did not have epidurals. Request for postoperative epidural analgesia was at the discretion of the attending pediatric cardiac anesthesiologist, in consultation with the attending pediatric cardiac surgeon. Median age at surgery was greater for patients with epidurals (6.7 [5.5–13.4] v. 5.1 [3.9–7.7] years, *p* = 0.022), as was weight at surgery (29.0 [23.1–48.5] vs. 18.4 [14.8–29.1] kg, *p* = 0.024). Although the age difference of 1.6 years is statistically significant, it may not be clinically significant as children in this age group have similar physiology. Sex distribution, preoperative anti-hypertensive use, mean upper extremity-lower extremity SBP gradient, systolic hypertension z-score, and aortic isthmus z-score did not differ between groups (Table 1).

### 3.2. Primary Outcome

**Figure 1:**
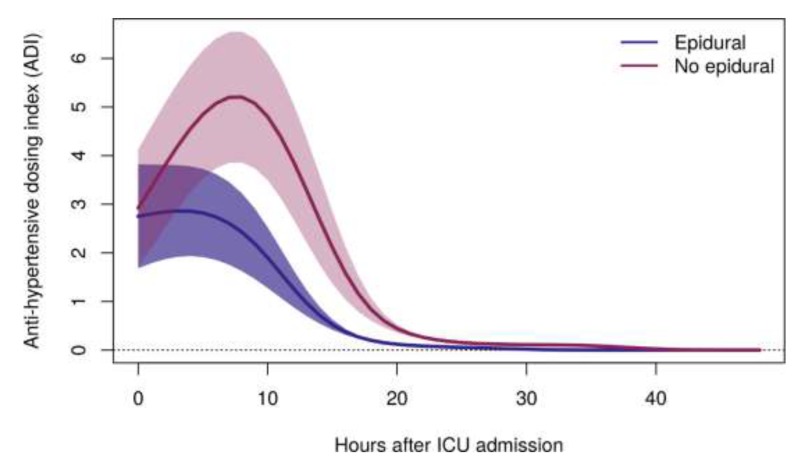
Mean ADI with 95% confidence band through 48 h after ICU admission for children undergoing coarctation repair via thoracotomy with and without epidurals, adjusted to the typical patient (age = 5.9 years; sex = male; weight = 23.1 kg; preoperative anti-hypertensive use = TRUE; preoperative systolic hypertension z-score = 2.95; preoperative SBP arm-leg gradient = 29 mm Hg; preoperative aortic isthmus z-score = −3.74). ADI = anti-hypertensive dosing index, a metric incorporating dose and duration of all anti-hypertensive infusions by determining the dose of each anti-hypertensive infusion, expressed as a multiple of the standard initial dose, multiplied by the total duration of each infusion at each dose; ICU = intensive care unit; SBP = systolic blood pressure. *p* < 0.001.

Children with epidurals had significantly decreased postoperative anti-hypertensive infusion requirement compared to children without epidurals (Table 2), as measured by total (cumulative) ADI (65.0 [28.5–130.3] v. 157.0 [68.6–214.7], *p* = 0.021) and average (mean area under the curve) ADI (49.0 [33.3–131.2] v. 163.0 [66.6–209.8], *p* = 0.01). After cumulative logit mixed-effects regression analysis, the time course of mean ADI was significantly altered among patients with epidurals (Figure 1), in whom mean ADI was decreased throughout the postoperative period compared to patients without epidurals (*p* < 0.001). Additional details on mean 

### 3.3. Secondary Outcomes

ICU length of stay (47.3 [30.8–53.2] v. 46.1 [29.6–59.2] hours, *p* = 0.927), hospital length of stay (3.96 [2.92–5.09] v. 3.77 [2.91–4.84] days, *p* = 0.6872), and number of patients discharged on anti-hypertensive medication (13/15 [86.7%] v. 24/31 [77.4%], *p* = 0.4591) were similar in patients with and without epidurals (Table 2), as was use of dexmedetomidine (6/15 [40.0%] v. 12/31 [39.0%], *p* = 0.9331). Nitroprusside was used significantly less often in patients with epidurals (9/15 [60.0%] v. 27/31 [87.0%], *p* = 0.0371). 

Epidural complications included pruritus treated with medication (3 patients), agitation prompting epidural removal on postoperative day one (1 patient), somnolence prompting adjustment of epidural dosing (1 patient), and transient orthostatic hypotension responsive to fluid bolus and discontinuation of furosemide (1 patient); no persistent hypotension or new neurologic deficits were noted. No patient required vasopressor or epidural dose adjustment for hypotension.

## 4. Discussion

This is the first study of children receiving an epidural for surgical repair of aortic coarctation via open thoracotomy. In this small observational retrospective cohort study at a single institution, patients with epidurals had decreased postoperative anti-hypertensive infusion requirement compared to patients without epidurals as measured by a novel parameter, the anti-hypertensive dosing index (ADI). This result is physiologically plausible: hypertension following coarctation repair is associated with sympathetic stimulation, and epidurals blunt sympathetic output. Retrospective analysis, however, cannot definitively assess causality. Improved analgesia might be as important a mediator of postoperative anti-hypertensive infusion requirement as epidural-induced sympathectomy, and other methods to minimize postoperative pain such as multimodal analgesia, other regional blocks, and minimally invasive surgical techniques might yield similar results. 

It is widely accepted that epidurals for open thoracotomy provide excellent analgesia and may improve postoperative respiratory status [45,46], potentially enhancing both patient/family satisfaction and clinical course. Consistent pain scores were not readily available for comparison between groups, preventing assessment of patient/family satisfaction. Although patients with epidurals had decreased postoperative anti-hypertensive infusions requirement compared to patients without epidurals, other markers of clinical course were similar.

Improved blood pressure control following coarctation repair has additional potential benefits. Aortic suture disruption and bleeding can be exacerbated by uncontrolled hypertension, a potentially fatal complication requiring prompt diagnosis and aggressive management [10]. Fortunately, reoperation for bleeding after surgical coarctation repair is now rare [47]. Post-coarctectomy syndrome is an uncommon complication including abdominal pain and distension, associated with reflex splanchnic vasoconstriction after restoration of pulsatile distal aortic flow [48]. Thoracic epidurals have been shown to induce splanchnic vasodilation [49] and could theoretically help prevent or treat post-coarctectomy syndrome. Our study was neither designed nor powered to assess effects of epidurals on such uncommon complications.

In an era of ever-increasing cost-conscious resource utilization, interventions to reduce ICU and hospital length of stay are of significant potential value. Although associated with decreased postoperative anti-hypertensive infusion requirement, epidurals in this study were not associated with decreased ICU or hospital length of stay. Multiple factors other than blood pressure control likely influenced transfer from the ICU and discharge from the hospital. This study does provide some reassurance that epidurals for coarctation repair in children are not associated with increased ICU or hospital length of stay, a common concern among providers at our institution. 

This study has several limitations. The cohort was small, and the analysis retrospective. Although patient groups were similar in many respects, and we adjusted for multiple covariables, residual confounding is always possible. The association between epidurals for coarctation repair in children and decreased postoperative anti-hypertensive infusion requirement is physiologically plausible, but retrospective analysis cannot definitely assess causality. The primary outcome, the ADI, is a composite metric incorporating dose and duration of all postoperative anti-hypertensive infusions, analogous to a vasoactive-inotropic score assessing blood pressure support [50], with the addition of a temporal element to account for infusion duration. The ADI is a novel metric that has not been validated as a marker of severity of hypertension. We used ADI rather than actual blood pressure, as anti-hypertensive regimens would be titrated to age-appropriate end-points. The ADI as a calculated score is a surrogate for clinical patient status. 

## 5. Conclusions

In this small, single-institution, observational retrospective cohort study, epidurals for coarctation repair in children were associated with decreased postoperative anti-hypertensive infusion requirement. Epidurals were not associated with length of ICU or hospital stay, or with discharge on anti-hypertensive medication. No significant epidural complications were noted. Prospective study of larger populations will be necessary to confirm these associations, address causality, verify safety, and assess other effects. 

## Figures and Tables

**Table 1:** Baseline characteristics.

Characteristic	Epidural	No Epidural	Test Statistic
Number of Patients (N)	15	31	N/A
Median Age = Years [IQR]	6.7 [5.5, 13.4]	5.1 [3.9, 7.7]	* *p* = 0.022 ^2^
Male Patients = N (%)	9 (60.0%)	20 (64.5%)	*p* = 0.766 ^1^
Median Weight = Kg [IQR]	29.0 [23.1, 48.5]	18.4 [14.8, 29.1]	* *p* = 0.024 ^2^
Pre-Op Anti-HTN Med = N (%)	9 (60.0%)	12 (38.7%)	*p* = 0.174 ^1^
Systolic HTN z-Score = Median score [IQR]	2.90 [1.45, 3.30]	3.00 [1.90, 3.20]	*p* = 0.550 ^2^
SBP Arm-Leg Gradient = mmHg [IQR]	23.0 [18.5, 33.5]	32.0 [20.8, 40.8]	*p* = 0.121 ^2^
Aortic Isthmus z-Score = Median Score [IQR]	−3.60 [−3.90, −2.25]	−3.80 [−4.36, −3.00]	*p* = 0.403 ^2^

Baseline characteristics of children undergoing coarctation repair via thoracotomy with and without epidurals. HTN = hypertension; IQR = interquartile range; SBP = systolic blood pressure. ^1^ Pearson test; ^2^ Wilcoxon test; * *p* < 0.05.

**Table 2:** Outcomes.

Outcome	Epidural	No Epidural	Test Statistic
Cumulative ADI = Median [IQR]	65.0 [28.5, 130.3]	157.0 [68.6, 214.7]	* *p* = 0.021 ^2^
Mean ADI = Median [IQR]	49.0 [33.3, 131.2]	163.0 [66.6, 209.8]	* *p* = 0.01 ^2^
ICU Length of Stay = Hours [IQR]	47.3 [30.8, 53.2]	46.1 [29.6, 59.2]	*p* = 0.927 ^2^
Hospital Length of Stay = Days [IQR]	3.96 [2.92, 5.09]	3.77 [2.91, 4.84]	*p* = 0.687 ^2^
Discharge on Anti-HTN Med = N (%)	13 (86.7%)	24 (77.4%)	*p* = 0.459 ^1^
Dexmedetomidine Used = N (%)	6 (40.0%)	12 (39.0%)	*p* = 0.933 ^1^
Nitroprusside Used = N (%)	9 (60.0%)	27 (87.0%)	* *p* = 0.037 ^1^

Outcomes for children undergoing coarctation repair via thoracotomy with and without epidurals. ADI = anti-hypertensive dosing index, a metric incorporating dose and duration of all anti-hypertensive infusions by determining the dose of each anti-hypertensive infusion, expressed as a multiple of the standard initial dose, multiplied by the total duration of each infusion at each dose; HTN = hypertension; IQR = interquartile range. ^1^ Pearson test; ^2^ Wilcoxon test; * *p* < 0.05.
